# Полегание пшеницы: генетические и экологические факторы и способы преодоления

**DOI:** 10.18699/VJ20.628

**Published:** 2020-07

**Authors:** E.V. Ageeva, I.N. Leonova, I.E. Likhenko

**Affiliations:** Siberian Research Institute of Plant Production and Breeding – Branch of the Institute of Cytology and Genetics of Siberian Branch of the Russian Academy of Sciences, Novosibirsk, Russia; Institute of Cytology and Genetics of Siberian Branch of the Russian Academy of Sciences, Novosibirsk, Russia; Siberian Research Institute of Plant Production and Breeding – Branch of the Institute of Cytology and Genetics of Siberian Branch of the Russian Academy of Sciences, Novosibirsk, Russia

**Keywords:** wheat, lodging, stem, Rht genes, lignin, anatomical and morphological characters, пшеница, полегание, стебель, гены Rht, лигнин, анатомо-морфологические признаки.

## Abstract

Полегание является одной из основных проблем снижения урожайности и качества зерна озимой и яровой пшеницы. Устойчивость этой культуры к полеганию в значительной степени зависит от факторов внешней среды, биологических и морфологических особенностей стебля и корневой системы. Селекция сортов на устойчивость к полеганию актуальна во многих странах мира, и в данном направлении получен ряд достижений. Высота растений – важный морфологический признак, связанный с устойчивостью к полеганию. Основным направлением для снижения риска возникновения полегания стало выведение сортов, несущих гены короткостебельности (Rht). Гены Rht-B1b, Rht-D1b, Rht8, Rht11 получили широкое распространение во всем мире среди сортов мягкой пшеницы благодаря значительному влиянию на хозяйственно ценные признаки, включая полегание. Немаловажным оказалось изучение анатомо-морфологических особенностей и химического состава тканей стебля, которые дополняют оценку устойчивости к полеганию и позволяют более полно характеризовать изучаемый сортовой материал. Особенно большую роль в прочности стебля многие исследователи отводят толщине стенок междоузлий и их анатомическому строению. Диаметр соломины, ее толстостенность и вес, большое количество сосудистых пучков и широкое кольцо механических тканей коррелируют с устойчивостью к полеганию. Важными структурными компонентами, обеспечивающими прочность стебля у пшеницы, являются содержание лигнина, кремния и целлюлозы. Большое значение в выявлении генетической основы взаимоотношений между анатомическими и морфофизиологическими признаками стебля и корневой системы и полеганием имеют молекулярно-генетический анализ и картирование генов и локусов количественных признаков. Генетические факторы, отражающие корреляции между полеганием и толщиной стенки стебля, числом проводящих пучков и другими параметрами, были картированы в хромосомах 1А, 1B, 2A, 2D, 3A, 4B, 4D, 5A, 5D, 6D и 7D. Установлено, что локусы с высоким фенотипическим эффектом в отношении толерантности к полеганию колокализуются с локусами, ответственными за высоту растения, диаметр и прочность стебля. Для повышения устойчивости к полеганию необходимы разработка комплекса агротехнических методов, снижающих влияние почвенноклиматических факторов, и создание толерантных к полеганию сортов.

## Abstract (en)

Lodging is one of the main factors in reducing the yield and grain quality of winter and spring wheat varieties. The resistance of wheat cultivars to lodging largely depends on environmental factors, biological and morphological features of the stem and root systems. Selection of the varieties for resistance to lodging is relevant in many countries of the world and has a number of achievements. Plant height is one of the most important morphological characters associated with lodging resistance. Breeding of the varieties carrying the dwarfing genes (Rht) is the main direction to reduce the risk of lodging. The Rht-B1b, Rht-D1b, Rht8 and Rht11 genes are widely used throughout the world due to their significant influence on agronomically valuable traits, including lodging. It turned out to be important to study the anatomical and morphological features and chemical composition of stem tissues, which complement the assessment of resistance to lodging and allow the varietal material to be more fully characterized. The thickness of stem internodes and their anatomical structure play an important role in the stem strength. The diameter of the stem, its thickness and weight, a large number of vascular bundles and a wide ring of mechanical tissues correlate with resistance to lodging. The content of lignin, silicon and cellulose are important structural components and provide the stem strength of wheat plants. Molecular genetic analysis and mapping of genes and quantitative trait loci are of great importance in identifying the genetic basis of the relationship between the anatomical and morphophysiological characters of the stem and root system and lodging. Genetic factors reflecting correlations between the lodging and the thickness of the stem wall, the number of vascular bundles and other characters were mapped to chromosomes 1A, 1B, 2A, 2D, 3A, 4B, 4D, 5A, 5D, 6D and 7D. It has been found that loci with high phenotypic effects on lodging tolerance are colocalized with loci responsible for plant height, stem diameter and stem strength. To increase resistance to lodging, it is necessary to develop a set of agrotechnical methods that reduce the influence of soil and climatic factors and create wheat varieties tolerant to lodging.

## Введение

Полегание – один из главных факторов, влияющих на урожайность пшеницы, качество зерна и содержание в нем питательных веществ. При полегании понижается устойчивость растений к болезням, формируется щуплое зерно, возникают проблемы с уборкой урожая. Полегание пшеницы в предуборочный и уборочный периоды, сопровождающиеся неблагоприятными погодными условиями и частым выпадением дождей, влечет за собой прорастание зерна на корню и значительно снижает хлебопекарные качества. Формирование мелкого зерна с низкой массой 1000 зерен в результате нарушения транспортировки ассимилянтов в колос – частое явление при полегании, наступившем до налива зерна (Berry et al., 2004; Packa et al., 2015; Khobra et al., 2019). Потери зерна на полегающих посевах озимой и яровой пшеницы составляют в среднем от 20 до 50 %. Полегание посевов на стадиях колошения, молочной, восковой и полной спелости зерна приводит к снижению урожайности на 31, 25, 20 и 12 % соответственно (Weibel, Pendleton, 1964; Fischer, Stapper, 1987). Даже кратковременное полегание растений вызывает потери урожая (Иванов, Дохунаев, 1979). Исследования А.Н. Лубнина (2006) показали наличие прямой средней зависимости (r = 0.346±0.08) между устойчивостью к полеганию и урожайностью; при этом в годы со слабым увлажнением эта связь выражена слабее. Чем устойчивее стебель, тем лучше развит колос: он крупнее, лучше озернен и имеет большой вес зерен. В полеглом ценозе у растений активнее развиваются листостеблевые болезни (мучнистая роса, бурая и стеблевая ржавчина, септориоз) и корневые гнили

Анализ многочисленных работ (Носатовский, 1965; Терентьев, 1974; Иванов, Дохунаев, 1979; Лелли, 1980; Захаров и др., 2014; Packa et al., 2015; Shah et al., 2017; Khobra et al., 2019) позволяет сделать заключение, что полегание – это по существу физиологическая реакция растений на определенные условия внешней среды: недостаток света, структуру почвы, ее избыточную влажность, влажный с высокой температурой микроклимат воздуха, высокое содержание азота и других минеральных составляющих (Mavi et al., 2004; Dahiya et al., 2018). Перечисленные факторы внешней среды считаются основными причинами полегания зерновых культур. Немаловажную роль играют климатические и погодные условия, в том числе скорость ветра, дожди и град. В подтверждение такого вывода говорят факты массового распространения полегания во влажных районах с обильными естественными осадками (Niu et al., 2016).

Устойчивость к полеганию зависит также от комплекса взаимосвязанных признаков, таких как анатомические и морфологические особенности стебля и корневой системы, биохимические и физиологические процессы, протекающие в организме растения. Согласно проведенным исследованиям, основными характеристиками, которые определяют устойчивость пшеницы к полеганию, являются: высота растения, длина и толщина стебля, размеры верхнего и нижнего междоузлий, число продуктивных побегов, диаметр и число проводящих пучков, содержание лигнина и целлюлозы в стебле, содержание растворимых сахаров, вес зерна в колосе (Kelbert et al., 2004; Ионова, 2009; Berry, 2012; Packa et al., 2015; Xiao et al., 2015; Khobra et al., 2019; Shah et al., 2019).

Последние достижения в разработке методов биотехнологии позволили идентифицировать ряд генов и локусов количественных признаков (QTL), контролирующих признаки, связанные с полеганием. С использованием двуродительских картирующих популяций были локализованы генетические факторы, ассоциированные с толерантностью к полеганию. Многие из них картированы в тех же геномных районах, где локализованы гены, отвечающие за высоту растения, диаметр и толщину стебля, урожайность (Verma et al., 2005; Kong et al., 2013; Berry P., Berry S., 2015). Сочетание методов полногеномного поиска ассоциаций и высокопроизводительного фенотипирования с использованием обширных коллекций сортов выявило новые геномные районы, коррелирующие с устойчивостью к полеганию (Kaur et al., 2017; Singh et al., 2019).

Селекция зерновых культур на продуктивность неразрывно связана с селекцией на устойчивость растений к неблагоприятным факторам внешней среды, включая устойчивость к полеганию. В настоящем обзоре рассмотрена роль различных факторов, влияющих на устойчивость пшеницы к полеганию, в том числе приведены результаты исследований по молекулярно-генетическому картированию генетических локусов, ассоциированных с проявлением данного признака.

## Типы полегания

Под полеганием принято понимать смещение стебля или всего растения от его вертикального положения. На практике различают прикорневое и стеблевое полегание пшеницы (Носатовский, 1965; Захаров и др., 2014; Khobra et al., 2019). Проявление корневого или стеблевого типа полегания зависит от характеристик конкретного сорта и факторов внешней среды. Прикорневое полегание связано с недостаточной механической прочностью самих корней либо недостаточно прочным сцеплением корневой системы с почвой (Packa et al., 2015; Shah et al., 2019). Архитектура корневой системы играет важную роль в поддержании устойчивости растения к полеганию. Известно, что глубина залегания корней, длина и плотность корневых волосков, угол расположения базальных корней и их изгиб влияют на устойчивость (Pinthus, 1967; Crook, Ennos, 1993). Одной из причин слаборазвитой корневой системы может быть дефицит влаги в почве в первую половину вегетации. Кроме того, прикорневое полегание встречается в районах с большим количеством осадков в фазы кущения и выхода в трубку, а также при орошении. Как правило, у генотипов с таким типом полегания прочный стебель, их анатомические показатели вполне соответствуют показателям соломины хорошо устойчивых к полеганию форм (Иванов, Дохунаев, 1979; Лелли, 1980). В результате переувлажнения верхнего слоя почвы под тяжестью надземной массы растений наблюдается смещение и растяжение корней, а иногда и их разрыв. Стебли растений теряют вертикальное положение и полегают. Более широкий угол распространения корневой системы снижает риск возникновения прикорневого полегания (Носатовский, 1965; Berry, 2012; Packa et al., 2015).

Стеблевое полегание пшеницы происходит под влиянием нагрузки на стебель, которая возрастает с увеличением веса на колос и удлинением соломины. Другими словами, происходит изгиб и даже слом соломины, преимущественно в районе второго и третьего междоузлия снизу (Терентьев, 1974; Zuber et al., 1999; Packa et al., 2015; Khobra et al., 2019). Особенно часто стеблевое полегание встречается у сортов с длинным и тонким стеблем. Наибольший вес колоса отмечается в конце спелости. Несмотря на то что третье нижнее междоузлие обладает меньшей устойчивостью соломины на излом, чем второе, в силу большей нагрузки, приходящейся на последнее, полегание пшеницы чаще всего отмечается именно во втором междоузлии. Увлажнение колоса увеличивает нагрузку на стебель, поэтому дожди в это время наиболее опасны для полегания пшеницы. Условия внешней среды приобретают особое значение во время формирования второго и третьего междоузлия. 

## Факторы, вызывающие полегание

Факторы, вызывающие полегание, можно подразделить на четыре группы: особенности самих растений (строение и свойства стебля, развитие и строение корней и др.); физические факторы (ветер, дождь, град, температура и световой режим и др.); агротехнические факторы (избыточное увлажнение и питание, в первую очередь азотное, недостаток фосфора и калия, завышенные нормы посева и др.); поражение пшеницы болезнями, которое в немалой степени связано с сортовыми особенностями и агротехникой.

Особое значение имеет выпадение осадков, а точнее не количество выпавших осадков, а интенсивность дождя и само его формирование. Как правило, сильный и порывистый ветер с дождем или после дождя является основной причиной полегания. Немаловажный фактор – загущенность посевов, которая приводит к возникновению взаимозатенения, в результате чего формируются тонкие и длинные стебли со слаборазвитой корневой системой (Иванов, Дохунаев, 1979; Foulkes et al., 2011).

Высокий уровень плодородия и структура почв тоже способствуют полеганию. Доступный для растения азот может поступать в него в результате минерализации растительных остатков или через минеральные удобрения (Berry et al., 2004). Применение минеральных удобрений ведет к мощному вегетативному росту пшеницы, к сильной кустистости, что влечет повышенную плотность агроценоза растений. Повышается конкуренция за пространство, свет и питательные вещества, что в конечном итоге приводит к формированию растений с более тонкими и длинными и более слабыми стеблями с меньшим количеством сухого вещества в нижней части междоузлия. Помимо удлинения стебля, наблюдается формирование тонких корней со слабой силой сцепления с почвой (Berry et al., 2004; Foulkes et al., 2011). Большое количество азота снижает также содержание целлюлозы и лигнина. Удобрения с калием, фосфором и микроэлементами оказывают меньшее воздействие на изменение прочности стебля, чем азотные, хотя эти данные неоднозначны (Mulder, 1954; Zhang et al., 2017; Khobra et al., 2019).

В современном производстве применяют интенсивные технологии возделывания пшеницы: внесение минеральных удобрений с целью получения высоких урожаев и повышения качества зерна. Поскольку применение минеральных удобрений может привести к полеганию, одним из способов решения данной задачи стало использование регуляторов роста (ретардантов), тормозящих процессы роста растения. Оказалось, что своевременное торможение роста вегетативных органов растений может способствовать развитию у них ряда полезных хозяйственных признаков: расширение пластинок листьев, повышение интенсивности их зеленой окраски, рост объема корневой системы, уменьшение продолжительности покоя семян, повышение всхожести и увеличение энергии прорастания. При этом важно, что сам ход физиологических процессов, определяющих продукционную способность обработанных растений, не должен претерпевать существенных изменений. Данные о влиянии ретардантов на физиологические функции растений противоречивы, однако большинство результатов свидетельствует, что регуляторы роста растений не оказывают отрицательного воздействия на фотосинтез, ограничивают чрезмерный расход воды и обеспечивают более благоприятный водный режим (Шаповал и др., 2010; Souza et al., 2010).

## Анатомические и морфофизиологические параметры стебля

Длина стебля – один из наиболее важных морфологических признаков, коррелирующий с устойчивостью к полеганию. Значительная склонность к полеганию у пшеницы наблюдается при высоте растения свыше 120 см (Дорофеев и др., 1976; Packa et al., 2015). Генотипы, имеющие укороченную соломину, отличаются толерантностью к полеганию и большей урожайностью зерна. Активное выведение короткостебельных сортов началось в 1960– 1970-х гг. при использовании в селекции различных аллелей генов Rht (Reduced height). Согласно каталогу генных символов, у пшеницы обнаружено более 40 аллелей генов Rht, локализованных в хромосомах второй и четвертой гомеологических групп и в хромосомах 5А, 5D, 6A, 7A, 7B (McIntosh et al., 2013, Supplements 2014–2017). Гены Rht условно делятся на две группы по их реакции на гиббереллиновую кислоту. Нечувствительные к гиббереллинам гены Rht1 и Rht2 картированы в коротких плечах хромосом 4B и 4D. Гены, чувствительные к гиббереллинам, были локализованы в хромосомах 2А, 2DS, 7BS и 5A. Кроме генов с постоянными символами, практически во всех хромосомах пшеницы картировано большое число QTL (Wurschum et al., 2017).

Несмотря на многочисленное число генов и аллелей Rht, только четыре из них – Rht-B1b (4BS), Rht-D1b (4DS), Rht8 (2DL) и Rht11 (Rht-B1e) – получили практическое применение при создании новых сортов (Knopf et al., 2008; Pearce et al., 2011; Xiao et al., 2015). Присутствие этих генов в сортах приводит к уменьшению числа и размеров междоузлий, сокращению длины колеоптиля, снижению длины стебля на 14–17 % и способствует увеличению урожайности до 20 % (Berry, 2012). Аллель Rht-B1e стимулирует существенно большее снижение высоты растений и увеличение урожайности по сравнению с аллелем Rht-B1b (Дивашук и др., 2012; Коршунова и др., 2014). На сегодняшний день более 70 % сортов в мире содержат по крайней мере один из этих Rht генов (Evans, 1998; Borojevic, Borojevic, 2005; Коршунова и др., 2014; Shah et al., 2019). Остальные локусы Rht не используются из-за негативных эффектов на урожайность и другие хозяйственно важные признаки (Daoura et al., 2014; Wang et al., 2014; Li et al., 2015).

В настоящее время известно, что гены Rht-B1b и Rht-D1b пшеницы кодируют мутантные белки DELLA, которые при образовании комплекса с гиббереллином и рецепторным белком репрессируют гиббереллиновый сигнал, влияя таким образом на рост и развитие растения (Peng et al., 1999; Yamaguchi, 2008; Pearce et al., 2011; Thomas, 2017). Мутации в структуре белков DELLA, снижающие чувствительность к действию гиббереллина, были выявлены у многих видов растений и детально изучены на примере арабидопсиса и риса (Билова и др., 2016; Vera-Sirera et al., 2016). Что касается пшеницы, то на данный момент молекулярные и биохимические функции мутантных белков DELLA недостаточно изучены, что серьезно затрудняет перспективы использования таких мутаций на практике.

Помимо длины, немаловажную роль играют другие параметры стебля. Установлено, что диаметр соломины, ее толстостенность и вес, количество сосудистых пучков и широкое кольцо механических тканей коррелируют с устойчивостью к полеганию (Емельянова, Резниченко, 1970; Иванов, Дохунаев, 1979; Shah et al., 2017). Особенную роль в прочности стебля многие исследователи отводят толщине стенок междоузлий и их анатомическому строению. Увеличение диаметра и толщины стебля приводит к повышению его прочности (Packa et al., 2015; Shah et al., 2017). Толщину стенок соломины обеспечивают клетки основной и механической тканей, а также компоненты проводящей системы. Толщина стебля пшеницы является ценным признаком, маркирующим потенциальную продуктивность растений (Лазаревич, 1999; Packa et al., 2015). Увеличение диаметра стебля способствует снижению риска возникновения полегания. Достигается это за счет увеличения диаметра проводящих пучков паренхимы и толщины склеренхимной ткани, которая, в свою очередь, зависит от числа слоев клеток и их диаметра (Лазаревич, 1999).

Имеется ряд исследований по выявлению генетических локусов, ассоциированных с параметрами стебля. С использованием дигаплоидной (DH) популяции озимой мягкой пшеницы шесть QTL для признаков «прочность стебля», «толщина стенки стебля», «диаметр сердцевины» и «диаметр стебля» идентифицированы в хромосомах 1А, 2D, 3B (Hai et al., 2005). В других работах генетические локусы, отражающие корреляции между полеганием и толщиной стенки стебля, были картированы в хромосомах 2A, 3A, 5A, 1B, 4B, 4D, 6D и 7D (Keller et al., 1999; Xiao et al., 2015). Для числа проводящих пучков были зарегистрированы QTL в хромосомах 1A, 2D, 5D и 7D (Shah et al., 2017). Результаты картирования также свидетельствуют, что локусы с высоким фенотипическим эффектом в отношении толерантности к полеганию колокализуются с QTL, которые ответственны за высоту растения, диаметр и прочность стебля (Berry P., Berry S., 2015; Li et al., 2015).

Для изучения генетической архитектуры такого сложного признака, как полегание, необходимы масштабные фенотипические и генотипические эксперименты с использованием популяций большого размера. По последним данным (Singh et al., 2019), генетическая основа полегания была исследована с помощью нового методического подхода высоковоспроизводительного фенотипирования (HTP, high-throughput phenotyping). Применение методов геномной селекции и геномного прогнозирования позволило идентифицировать ключевой геномный район в хромосоме 2А и минорные локусы в других хромосомах, совпадающие по локализации с QTL, картированными ранее в других исследованиях.

С помощью современной микроскопической техники установлено, что прочность стебля обеспечивается комплексом анатомических признаков: численностью и взаимным расположением проводящих пучков, топографическим положением в стебле механических тканей и их параметрами (Лазаревич, Мыхлык, 2014). Выполненная часть стебля представлена эпидермисом, первичной корой и центральным цилиндром, состоящим из периферического кольца склеренхимы, проводящих пучков и запасающей паренхимы. В сердцевине паренхимы у мягкой пшеницы имеется полость – медуллярная лакуна, которая образуется в результате разрушения сердцевины при удлинении стебля (Носатовский, 1965; Емельянова, Резниченко, 1970). 

Проводящие пучки паренхимы и первичной коры вместе составляют проводящую систему растения и одновременно входят в состав механической ткани. При изгибе или ломкости стебля в результате полегания происходит повреждение сосудистых проводящих пучков. Несмотря на отсутствие однозначных достоверных корреляций между числом проводящих пучков и устойчивостью к полеганию, при изучении анатомической структуры стебля видов и сортов яровой пшеницы, различающихся по полеганию, отмечена разница в количестве сосудистоволокнистых пучков в междоузлиях (Ageeva et al., 2019). Увеличенное число сосудистых пучков наблюдалось у сортов, устойчивых к полеганию. 

По мнению С.В. Лазаревича (1999), устойчивости к полеганию способствует ритмическое чередование крупных и малых пучков и наличие у крупных пучков склеренхимных обкладок. В случае узкого слоя склеренхимы формируются растения с тонким и ломким стеблем.

Устойчивость к полеганию представляет собой комплекс тесно взаимодействующих между собой признаков, поэтому важен не только широкий слой склеренхимной ткани, но и большое содержание в нем лигнина и целлюлозы (Shah et al., 2017). На сегодняшний день отсутствует достаточная информация о генетическом контроле содержания целлюлозы у пшеницы, за исключением результатов S. Kaur с коллегами (Kaur et al., 2017), которые с помощью полногеномного поиска ассоциаций выявили девять маркеров SNP, с высокой достоверностью связанных с генами, кодирующими β-тубулин, ауксин-индуцируемый белок и трансмембранный белок с неизвестной функцией. Предполагается, что эти гены могут быть вовлечены в биосинтез целлюлозы и влиять на прочность стебля.

Лигнин – важный структурный компонент вторичной клеточной стенки, который связан и с ростом растения, и с прочностью стебля. У пшеницы наблюдается значительная корреляционная связь между устойчивостью к полеганию и содержанием лигнина в стебле. D. Peng с коллегами в своем исследовании установили, что сорта с высоким содержанием лигнина могут быть использованы в качестве источников с целью создания образцов, толерантных к полеганию (Peng et al., 2014). Кроме того, в совокупности увеличение содержание лигнина и гемицеллюлозы увеличивает прочность стебля. Сорта с низким содержанием этих компонентов более склонны к полеганию (Zheng et al., 2017).

Еще одним важным структурным компонентом, обеспечивающим прочность стебля у пшеницы, является кремний (Иванченко, Резанова, 2016; Shah et al., 2017). Биологический активный кремний укрепляет эпидермис, кору и сосудисто-проводящие ткани, тем самым существенно повышая пластичность, упругость, прочность стебля, листьев и защитные функции растений (Иванченко, Резанова, 2016). Это происходит за счет того, что кремний значительно увеличивает содержание целлюлозы и лигнина в клетках склеренхимы. Он накапливается в эпидермальных тканях и коронарных клетках, обеспечивая их механическую прочность и жесткость (Shah et al., 2017; Zhang et al., 2017; Khobra et al., 2019). В тканях зерновых культур двуокись кремния составляет более половины остальных микроэлементов, поглощаемых из почвы. Зерновые поглощают кремния в 10–20 раз больше, чем бобовые. В течение вегетационного периода количество кремния растет, достигая максимума к его завершению (Козлов и др., 2015; Walsh et al., 2018).

Отсутствие необходимого количества других микроэлементов также может оказывать влияние на прочность стебля. Дефицит фосфора вызывает снижение толщины стенки и физической прочности стебля. Этот элемент способствует укреплению корневой системы, играет главную роль в переносе энергии, в дыхании и фотосинтезе. Недостаток калия приводит к уменьшению диаметра стебля, поскольку он участвует в лигнификации клеточной стенки и колленхимы (Емельянова, Резниченко, 1970; Shah et al., 2017).

## Заключение

Таким образом, степень устойчивости к полеганию растений зависит от многих внешних и внутренних факторов. Для снижения риска возникновения полегания необходимо учитывать не только анатомо-морфологические и физиологические особенности сорта, такие как длина и толщина стебля, длина междоузлий, содержание лигнина и целлюлозы и др., но и климатические особенности регионов, на территории которых культивируются генотипы. Немаловажно учитывать такие параметры, как время и плотность посева, внесение удобрений и регуляторов роста растений. Связь полегания с высотой стебля и другими анатомо-морфологическими параметрами констатируется во многих научных работах, однако влияние большинства параметров пока до конца не изучено. В свете использования новых биотехнологических методов и результатов секвенирования генома пшеницы и других злаков необходимо продолжать работы по идентификации целевых локусов для установления более точной взаимосвязи между полеганием и другими агрономическими признаками. При осуществлении селекционного процесса, начиная с оценки и подбора родительских пар и заканчивая исследованием новых гибридных форм, следует комплексно подходить к проблеме, решая ее с применением всех возможных подходов в зависимости от особенностей места выращивания и уровня требований к получаемой продукции.

## Conflict of interest

The authors declare no conflict of interest.
